# Monitoring nurturing care environments for early childhood from the national to the municipal level

**DOI:** 10.1111/mcn.13327

**Published:** 2022-03-01

**Authors:** Rafael Pérez‐Escamilla, Sonia Venancio, Gabriela Buccini

**Affiliations:** ^1^ Department of Social and Behavioral Sciences Yale School of Public Health New Haven Connecticut USA; ^2^ Research Department Sao Paulo Health Institute Sao Paulo Brazil; ^3^ Department of Social and Behavioral Health University of Nevada Las Vegas Nevada USA

**Keywords:** early childhood development, decision making

## Abstract

- There is a dearth of indicators and management information systems to assess early childhood nurturing care environments.
- The Brazilian Early Childhood Care Friendly Municipal Index (IMAPI) is a robust tool to assess nurturing care environments at the local level.
- IMAPI was able to detect nurturing care inequities within and across regions.
- Further validation work is needed to confirm the utility of IMAPI for policymaking.

The Global Nurturing Care Framework developed from the 2016 Lancet Early Childhood Development Series (Black et al., 2017; Britto et al., 2017; Richter et al., 2017) indicates that children need five interrelated and indivisible care domains to reach their full potential. These are Good Health, Adequate Nutrition, Opportunities for Early Learning, Security and Safety, and Responsive Caregiving. Nurturing care begins since gestation and continues throughout the life course (Black et al., 2021). In 2018, the World Health Organization (WHO) and UNICEF adopted the Nurturing Care Framework (World Health Organization et al., 2018) as an evidence‐based road map for countries to follow to implement effective integrated Early Childhood Development (ECD) Programmes. However, efficient monitoring and evaluation of nurturing care environments (i.e., Good Health, Adequate Nutrition, Opportunities for Early Learning, Security and Safety, and Responsive Caregiving), are key for countries to be able to properly implement the Nurturing Care Framework and attain of the Sustainable Development Goals (SDGs) are strongly needed (Black et al., 2017; Britto et al., 2017; Richter et al., 2017; World Health Organization et al., 2018). Although previous efforts have attempted to develop such systems at the national and to some extent the regional or provincial level, this had not been previously attempted at the municipal level (Pedroso et al., 2021). This article introduces the supplement “Development and Application of the Brazilian Early Childhood Care Friendly Municipal Index” by summarising the history, methods, and applications of the Brazilian Early Childhood Care Friendly Municipal Index (IMAPI), and making recommendations on the way forward.

The IMAPI was developed following an 8‐step methodology. The first article of this supplement (Buccini et al., 2021a) describes the first three steps involved of an innovative participatory multisectoral decision‐making process to identify indicators across the five domains of the Nurturing Care Framework. The IMAPI indicators were selected following four distinct activities conducted with the IMAPI team, technical stakeholders, and experts (Buccini et al., 2021a). Detail on participants' institutions is described in Table S1. First, indicators across Nurturing Care Framework domains were identified through a comprehensive literature review conducted by the IMAPI team that included investigators who collectively had expertise in epidemiology, maternal–child nutrition, implementation science and data science, and machine learning, including the co‐authors of this article (GB, RPE, and SV). Second, four technical panels composed of stakeholders from federal, state, and municipal levels were consulted to identify data sources, their availability at the municipal level, and the strengths and weakness of each potential indicator. Third, national and international ECD experts participated in two surveys to score the expected performance of each nurturing care indicator, following the SMART indicator principles. Fourth, the IMAPI team reached a consensus on 31 nurturing care indicators across the five Nurturing Care Framework domains – Good health (*n* = 14), Adequate nutrition (*n* = 4), Opportunities for early learning (*n* = 7), Security and safety (*n* = 5), and Responsive caregiving (*n* = 1).

The second article of this supplement (Buccini et al., 2021b), describes the remaining steps of IMAPI's 8‐step methodology. Steps 4–6 consisted of statistical methods used to analyse and standardise the nurturing care indicators. In steps 7 and 8, the set of nurturing care indicators available at the municipal level in the Brazilian databases between 2015 and 2019 was summarised into an overall IMAPI score and sub‐scores representing the Nurturing Care Framework domains. Following the statistical criteria of having at least two indicators in the sub‐score domain to be included in the overall IMAPI score, the Responsive caregiving domain was excluded. The overall IMAPI score is composed of 30 indicators across four Nurturing Care Framework sub‐scores. The overall IMAPI score and sub‐scores ranged from 0 to 100, and scores were categorised into high, medium, and low categories based on the corresponding tercile distributions. The validity of the IMAPI scores was tested by assessing predictive and concurrent correlations with the Basic Education Development Index (IDEB, which summarises elementary‐aged children's school achievement) and the number of socially vulnerable children, respectively (Buccini et al., 2021b). As expected, there was a strong positive correlation between IMAPI scores and school achievement and a negative one between IMAPI and social vulnerability in children. Although this is encouraging, moving forward further validation analysis is needed to confirm that IMAPI correlates well with aggregated ECD outcomes at the municipal level.

Furthermore, in the second article (Buccini et al., 2021b), IMAPI was able to identify sociodemographic inequities by nurturing care domains. While low subscores in Good health, Adequate nutrition, and Opportunities for early learning were more frequent in municipalities in the North and Northeast, low subscores in Security and safety were more frequent in the Central‐West, closely followed by the South. High subscores in Good health and Security and safety were more frequent for very small population size municipalities; by contrast, high subscores in Adequate nutrition and Opportunities for early learning were more frequent in metropolitan areas. In addition, IMAPI was able to capture important between‐ and within‐region inequities. As expected, low IMAPI scores were more frequent in the North and Northeast regions and in small and medium‐size municipalities. Conversely, high IMAPI scores were more frequent in the more prosperous South and Southeast regions and in metropolitan areas. Between‐region analyses confirmed nurturing care environments inequities between the North/Northeast and South/Southeast. Lastly, the biggest within‐region inequities in IMAPI scores were found in the Northeast and the North. IMAPI distinguished the nurturing care ECD environments across Brazilian municipalities, therefore it has the potential to inform equitable and intersectoral multilevel decision making.

The third article of the supplement (Buccini et al., 2022), showcases how IMAPI can be used in a single metropolitan area to yield information with the potential to inform decisions regarding nurturing care investments. In this instance, IMAPI was applied in Brasilia, Brazil's capital with a large metropolitan population of 2,881,854 inhabitants living across 31 districts. Hence, IMAPI scores were estimated at the municipal level (IMAPI‐M, 31 indicators) and at the district level (IMAPI‐D, 29 indicators). A quantitative prioritisation process was developed to guide the identification of nurturing care indicators with low scores in each IMAPI analysis. The indicators selected were jointly mapped into three dimensions: First, the five domains of the Nurturing Care Framework (Black et al., 2017). Second, the six levels of the socioecological model of nurturing care that were developed by WHO (WHO et al., 2018) were adapted by the IMAPI team. The six levels of the socioecological model were operationalized as follows: *Enabling Policies*, indicators related to public policies that enable the nurturing care environment; *Empowered communities*, indicators that characterise the vulnerability of nurturing care within the communities; *Support Services*, indicators related to the availability of services and actions that directly impact ECD outcomes; *Caregivers' capabilities*, indicators related to caregivers' characteristics and their ability to provide nurturing care; *Family capabilities*, indicators related to families' characteristics and their ability to provide nurturing care; and *Child characteristics*, indicators related to the biological risk factors to ECD outcomes. Third, the role of indicators at assessing the enabling environment for nurturing care was developed by the IMAPI team after reviewing previous research that conceptualises the performance of health indicators in Brazil and other Latin American countries (Albuquerque & Martins, 2017; Pan American Health Organization, 2018) and were operationalized as follows: *Effort* reflects the effort of the municipal management in offering ECD‐related policies, programmes, and services; *Coverage* reflects the capacity of the ECD system to meet the demand of families for programmes and services; *Quality* reflects the quality of ECD‐related programmes and services, and *Result* reflects the effectiveness of ECD‐related programmes and services.

Out of 28 common nurturing care IMAPI indicators across IMAPI‐M and IMAPI‐D, the following four were prioritised in both analyses: “Coverage of information on child nutritional status” (Adequate nutrition, Supportive Services, Coverage), “Coverage of daycare and preschool” (Opportunities for early learning, Supportive Services, Coverage), “Number of students per preschool professional” (Opportunities for early learning, Caregivers' capabilities, Quality), and “Visits by national home‐visiting parenting skills programme” (Responsive caregiving, Enabling policies, Effort). The goal of mapping the three dimensions of nurturing care indicators (i.e., Nurturing Care Framework domain, level of socioecological, and role of nurturing care indicators) aimed to facilitate the interpretation of IMAPI findings and increase the understanding of decision‐makers about the causal pathways and interconnectedness within and across the Nurturing Care Framework domains. This study does indicate the IMAPI has a strong potential to inform ECD decision‐making in metropolitan areas that have some level of autonomy.

Lastly, the fourth article of the supplement (Pedroso et al., 2021), a global scoping review on existing methodologies to measuring nurturing care clearly shows the major gap that IMAPI fills as no previous nurturing care indices had been developed and tested for monitoring nurturing care environments at the municipal level. Given the successful development and testing of IMAPI the question is, what's next? In our view, there are two important steps that need to be taken to further advance the understanding and potential of municipal level nurturing care monitoring tools based on indicators such as IMAPI.

First, quantitative studies are needed to examine the correlation between municipal IMAPI scores and ECD outcomes to fully confirm the predictivity validity of IMAPI. However, currently, ECD outcomes are not available at the populational level in Brazil, however this is likely to change soon with the ongoing application of PIPAS (*Primeira Infância para Adultos Saudáveis* – Early Childhood for Healthy Adults). PIPAS is a validated instrument that can be used to quickly and inexpensively evaluate developmental outcomes of infant and young children under five years of age during multi‐vaccination campaigns at the municipal level in Brazil (Venancio et al., 2021). In recent years, different instruments have been developed for monitoring ECD outcomes at the population level (McCoy et al., 2021); and international agencies have called for the development of an instrument that allows comparable measurement of ECD at a global level (Cavallera et al., 2019), yet no consensus exists on which is the best instrument to do so. In this context, PIPAS offers the possibility of obtaining ECD outcomes and additional information across the five domains of the Nurturing Care Framework, making it possible to analyse the influence of such domains on ECD, as recently demonstrated in Brazil (Venancio et al., 2021, 2022). Hence, IMAPI and PIPAS can be used in a complementary way, because while IMAPI uses existing secondary data to evaluate the nurturing care environments, PIPAS collects complementary information on ECD outcomes through population surveys. This information, together, could make it possible to assess the influence of multiple factors on nurturing care and how it impacts ECD in Brazil, from the national to the municipal level.

Second, qualitative research is needed to understand if an IMAPI driven qulaity assurance process could help improve nurturing care program coverage and quality at the municipal level. This approach has been followed with a breastfeeding decision making toolbox analogous to IMAPI but only at the national level (Buccini et al., 2019; Hromi‐Fiedler et al., 2019; Pérez‐Escamilla et al., 2018). For this reason, dmeonstrating how decision‐making at the municipal level can be improved when linked to an IMAPI monitoring system would be truly groundbreaking.

## CONFLICT OF INTERESTS

The authors declare no conflict of interests.

## AUTHOR CONTRIBUTIONS

RPE wrote the first draft that was critically reviewed by SV and GB. All authors read and approved the submitted version of the manuscript.

## Supporting information

Supporting information.

## Data Availability

Data sharing is not applicable to this article as no new data were created or analysed in this study.
